# Multi-granularity contrastive learning model for next POI recommendation

**DOI:** 10.3389/fnbot.2024.1428785

**Published:** 2024-06-14

**Authors:** Yunfeng Zhu, Shuchun Yao, Xun Sun

**Affiliations:** ^1^Suzhou Industrial Park Institute of Service Outsourcing, Suzhou, China; ^2^School of Computer Engineering, Suzhou Vocational University, Suzhou, China

**Keywords:** multi-granularity information, graph convolutional networks, self-attention networks, contrastive learning, POI recommendation

## Abstract

Next Point-of-Interest (POI) recommendation aims to predict the next POI for users from their historical activities. Existing methods typically rely on location-level POI check-in trajectories to explore user sequential transition patterns, which suffer from the severe check-in data sparsity issue. However, taking into account region-level and category-level POI sequences can help address this issue. Moreover, collaborative information between different granularities of POI sequences is not well utilized, which can facilitate mutual enhancement and benefit to augment user preference learning. To address these challenges, we propose multi-granularity contrastive learning (MGCL) for next POI recommendation, which utilizes multi-granularity representation and contrastive learning to improve the next POI recommendation performance. Specifically, location-level POI graph, category-level, and region-level sequences are first constructed. Then, we use graph convolutional networks on POI graph to extract cross-user sequential transition patterns. Furthermore, self-attention networks are used to learn individual user sequential transition patterns for each granularity level. To capture the collaborative signals between multi-granularity, we apply the contrastive learning approach. Finally, we jointly train the recommendation and contrastive learning tasks. Extensive experiments demonstrate that MGCL is more effective than state-of-the-art methods.

## 1 Introduction

Location-based social networks (LBSNs), a new type of social media, such as Yelp and Foursquare, are typical LBSNs applications. As a result, a large amount of check-in data have been accumulated, which provides an excellent opportunity to understand users' mobile behaviors. The next POI recommendation predicts where a user will go next, providing mutual benefits for POI holders and users. Due to its highly practical value, the next POI recommendation has attracted extensive attention from academia and the industry community.

Recently, how to improve the performance of next POI recommendation has been extensively studied (Zhang and Chow, 2015; Wang et al., 2016; Zhao et al., 2019; Afzali et al., 2021). In the early stages, Markov Chain (MC) (Cheng C. et al., 2013; Cheng H. et al., 2013; Liu et al., 2013; He et al., 2016) and Matrix Factorization (MF) (Lian et al., 2014; Zhang et al., 2019; Davtalab and Alesheikh, 2021; Xu et al., 2023) were commonly employed to model sequential transitions in conventional POI recommendations, treating user behavior patterns as static. However, conventional methods tend to overlook the dynamic evolution of user preferences over time and face challenges in handling sparse sequential data. This limitation has prompted a shift toward neural network-based approaches, particularly with the emergence of deep learning (DL). In recent years, researchers have made a series of important breakthroughs based on the recurrent neural network (RNN) model. Innovative initiatives such as the spatiotemporal recurrent neural network (STRNN) have successfully integrated time and geographical context information into the model (Liu et al., 2016; Zhu et al., 2017; Fang and Meng, 2022; Wu et al., 2022). The key to these methods is to process time series data efficiently. In this research area, the subsequent studies by Liu et al. (2021) and Zhao et al. (2022) further extended the Long Short-Term Memory (LSTM) or Gated Recurrent Unit (GRU) model to better capture long-term and short-term dependencies (Zhao et al., 2018). This enh anced approach involves the introduction of specialized spatial and temporal gates to regulate the flow of contextual information. As self-attention networks (SAN) show great potential in process sequential tasks, SAN-based models such as SASRec (Kang and McAuley, 2018) and TiSASRec (Li et al., 2020), quickly surpassing the traditional convolutional neural network (CNN) or RNN-based methods and becoming an advanced model in the field of sequential recommendation. Recently, some SAN-based works have further improved the performance of next Point-of-Interest (POI) proposals by introducing hierarchical grids (Lian et al., 2020; Cui et al., 2021). This innovative approach aims to fully exploit geographic information while taking into account non-adjacent locations and non-contiguous visits, improving model performance by explicitly incorporating spatial and temporal proximity. Graph neural network (GNN) (Rao et al., 2022) and knowledge graph (KG) have garnered more attention on the next POI recommendation due to the ability to better express entity relationships (Rao et al., 2022; Wang et al., 2022a,b; Yang et al., 2022). This evolution in recommendation systems showcases a continuous effort to refine approaches for handling sequential data and improving the accuracy of POI recommendations.

**Figure 1:**
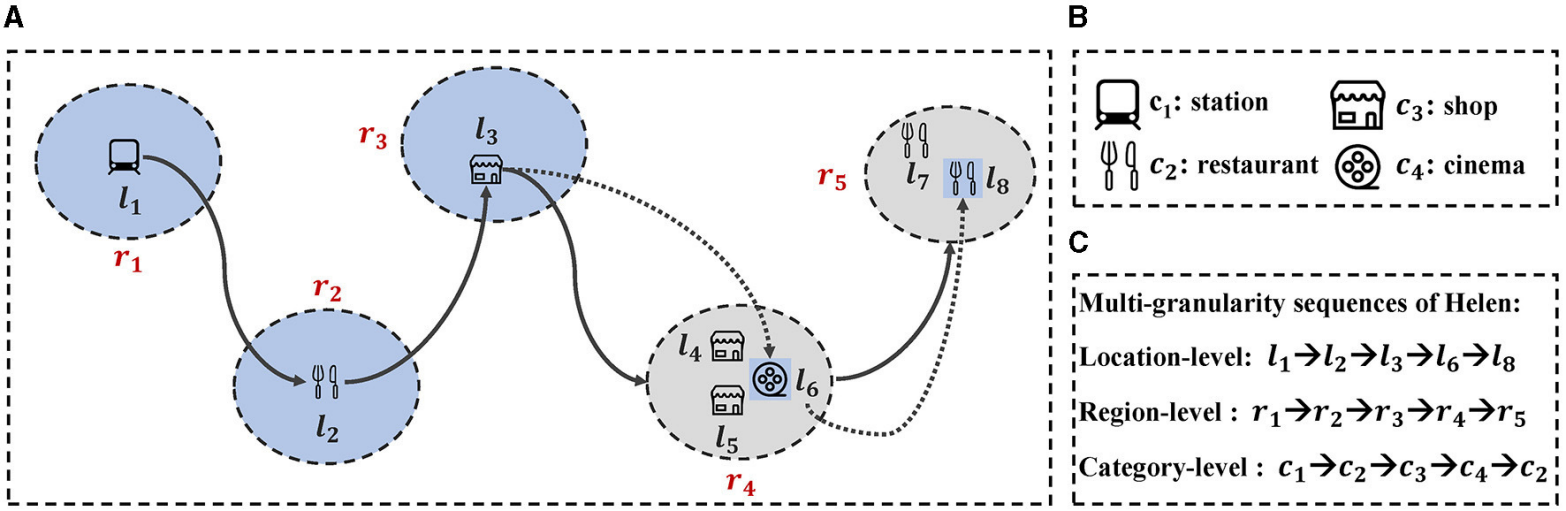
An example of Helen's multi-granularity POI sequences. **(A)** An illustration of Helen's movements across different locations and regions. Each circle represents a region, with arrows indicating the sequence of her movements. **(B)** Icons representing different categories of POIs. **(C)** Multi-granularity sequences of Helen's movements.

Although the above methods have achieved advanced performance, these methods still face the following issues. First, most existing studies exploit location-level POI sequences, ignoring the existence of region-level and category-level POI sequences. As illustrated in Figures 1A–C, Helen visited location-level POI at *l*_1_, *l*_2_, and *l*_3_ successively, but Helen may leave a rough footprint, e.g., region *r*_4_ instead of the precise POI *l*_4_, *l*_5_, and *l*_6_. The accessible sequence of check-ins will become: “*r*_1_→*r*_2_→*r*_3_→*r*_4_”. Thus, region-level POI are common and essential in real life. Finally modeling POI category labels are crucial for next POI recommendation as it improves accuracy and diversity. For example, if a user has visited museums and art galleries, and our model determines they are interested in art but not sure what type of place they want to visit, category label modeling becomes essential. Without it, we may recommend places they are not interested in, reducing satisfaction and usability.

Second, following the above innovations, most subsequent POI recommendation models adopted designs based on the supervised learning paradigm. The supervision signals of these models are mainly derived from user interaction data with POIs, but since the supervision signals are usually sparse, this may have an impact on the learning of user preferences. Existing work attempts to utilize supervised signals to enhance the quality of user preference learning. For example, CTLTR (Zhou et al., 2022) is a trip prediction model that uses self-supervised learning to capture supervised signals to enhance user preference learning. However, existing methods usually only use location-level POI check-in trajectories to mine supervised signals, while ignoring the supervised signals of region-level and category-level POI sequences.

To this end, we propose a multi-granularity contrastive learning (MGCL) model for next POI recommendation, which utilizes multi-granularity representation and contrastive learning to improve the next POI recommendation performance. Specifically, location-level POI graph, category-level, and region-level sequences are first constructed. Then, we use graph convolutional networks on POI graph to extract global cross-user sequential transition patterns. Then, self-attention networks are used to learn individual user sequential transition patterns for multi-granularity. To capture the collaborative signals among multi-granularity, we apply a contrastive learning approach, which uses pairwise contrastive learning at the location-level, region-level, and category-level representations. Finally, we joined learning the next POI recommendation task and the multi-granularity contrastive learning task. Through extensive experiments on real-world datasets, the MGCL model consistently outperforms current leading methods in all aspects. The main contributions of this study can be summarized as follows:

- To the best of our knowledge, this is the first work to apply contrastive learning for next POI recommendation, which can capture the collaborative signals among different granularities and facilitate mutual enhancement.
- We propose a framework called Multi-granularity Contrastive Learning for Next POI Recommendation (MGCL). To achieve better recommendation performance, we also adopt a multi-task learning approach.
- The effectiveness of the MGCL model was confirmed through experiments on three real-world datasets, confirming that our model has made significant progress in improving recommendation performance.

The subsequent sections of this study are structured as follows: In Section 2, we commence with a discussion of related work. Moving on to Section 3, we present our proposed model, MGCL, designed for next Point-of-Interest (POI) recommendation. Section 4 provides an overview of the experimental results. Lastly, in Section 5, we draw conclusions to summarize the study.

## 2 Related work

In this section, we undertake a comprehensive review of related work from two distinct perspectives: POI recommendation and contrastive learning.

### 2.1 POI recommendation

Next POI recommendation aims to learn the user preference transition patterns, as well as the spatio-temporal information relationship between user check-ins, time of check-ins, and geographical location. Due to its great commercial value, this task has attracted much attention. Most of the next POI recommendation methods are based on Markov Chain (MC) which focus on iteratively determining the transformation matrix of the next behavior or deep learning which processes the recommendation task in a data-driven manner. Specifically, factorization machines (FMs) (Rendle, 2010) suggest dealing with the non-adjacent check-in problem in the next POI recommendation, which is not easy to model with the MC-based methods. Then, Cheng C. et al. (2013) attempt to incorporate spatio-temporal information into existing models. Zhang et al. (2020b) propose a personalized geographical influence modeling method (PGIM) that jointly learns users' geographical and diversity preferences to improve POI recommendations, addressing limitations in spatial relevance and diversity in existing methods. Liu et al. (2018) propose a privacy-preserving framework using partially homomorphic encryption to design two protocols for trust-oriented POI recommendation. It proves that these protocols are secure against semi-honest adversaries and demonstrates through experiments that they achieve privacy preservation with acceptable computation and communication costs. Compared with MC-based methods, DL-based methods can usually achieve better performance.

Next POI recommendation methods based on early deep learning are RNN-based and their variants. STRNN (Liu et al., 2016) enhances the spatio-temporal modeling capability of RNN by using spatio and temporal intervals between successive check-ins. Time LSTM (Zhu et al., 2017) adds time information to the long and short memory networks, while STGN (Zhao et al., 2022) further integrates spatial information by designing space-time gates. Recently, with the development of Transformers, the attention mechanism has been widely used in the next POI recommendation. STAN (Luo et al., 2021) uses the self-attention network (SAN) to model long-term dependencies in long-term use check-in sequences. MGSAN (Li et al., 2021b) employs a multi-granularity representation along with a self-attention mechanism to characterize Point-of-Interest (POI) sequences at both individual and collective levels. This dual-level granularity enables the model to adeptly grasp behavior transition patterns, thereby enhancing recommendation performance. MCMG (Sun et al., 2022) utilizes a multi-channel encoder to capture multi-granularity sequential transition patterns, thereby improving recommendation performance. We argue that the collaborative signals among different granularities of POI sequences can facilitate each other and benefit augment user preference learning.

### 2.2 Contrastive learning

In recent years, contrastive learning (CL) (Chuang et al., 2020; Ho and Vasconcelos, 2020; Liu et al., 2023) has shown potential in solving data sparsity problems in Computer Vision (CV) (Chen et al., 2020), Graph/Node Classification (G/NC) (You et al., 2020), and Natural Language Processing (NLP) (Gao et al., 2021) areas. Contrastive learning methods have been explored by certain researchers in attempts to be applied to recommendation systems (Xie et al., 2022). For example, SGL (Wu et al., 2021) employs a strategy involving the random removal of edges, vertices, and random walking to create diverse perspectives of the initial graph. The aim is to maximize the consistency of identical nodes across these varied views. NCL (Lin et al., 2022) introduces users (or items) and neighbors from structural space and semantic space, respectively, and uses them as positive (or negative) contrastive pairs. To improve the graph contrastive learning in the recommendation, SimGCL (Yu et al., 2022) introduces a straightforward contrastive learning approach. In contrast to employing a graph augmentation mechanism, the method opts for the addition of uniform noise to the embedding space for generating contrasting views. CL4SRec (Xie et al., 2022) innovatively incorporates contrastive learning into sequential recommendation. It achieves this by introducing three random data augmentation strategies, which are employed to generate contrastive sequences based on the original sequences for the first time in this context. DuoRec (Qiu et al., 2022) engages in contrastive learning at the model level as a strategy to alleviate the degradation of representation. CTLTR (Zhou et al., 2022) is a trip prediction model that uses self-supervised learning to capture supervised signals to enhance user preference learning. However, existing methods usually only use location-level POI check-in trajectories to mine supervised signals, while ignoring the supervised signals of region-level and category-level POI sequences.

## 3 Problem statement

Let **𝒰** = {*u*_1_, *u*_2_, …, *u*_|𝒰|_}, **ℒ** = {*l*_1_, *l*_2_, …, *l*_|ℒ|_}, **ℛ** = {*r*_1_, *r*_2_, …, *r*_|ℛ|_}, **𝒞** = {*c*_1_, *c*_2_, …, *c*_|𝒞|_} represent the sets of users, locations (Points of Interest - POI), regions, and categories, respectively. A check-in track (*u, l, t, g, r, c* ) indicates that user *u* visited a POI *l* in region *r* at time *t*, where *l* is geocoded by *g* (longitude, latitude), and the category is *c*. The POI trajectory of user *u* is denoted as ${ℒ}^{u}=\left\{L_{t_{1}}^{u},L_{t_{2}}^{u},...,L_{t_{k}}^{u}\right\}$. The corresponding region and category check-in trajectories are denoted as ${ℛ}^{u}=\left\{R_{t_{1}}^{u},R_{t_{2}}^{u},...,R_{t_{k}}^{u}\right\}$ and ${𝒞}^{u}=\left\{C_{t_{1}}^{u},C_{t_{2}}^{u},...,C_{t_{k}}^{u}\right\}$. Given **ℒ**^*u*^, **ℛ**^*u*^, and **𝒞**^*u*^, our objective is to predict the next location *l*_*t*_*k*+1__ for user *u* at time *t*_*k*+1_.

## 4 The proposed methodology

**Figure 2:**
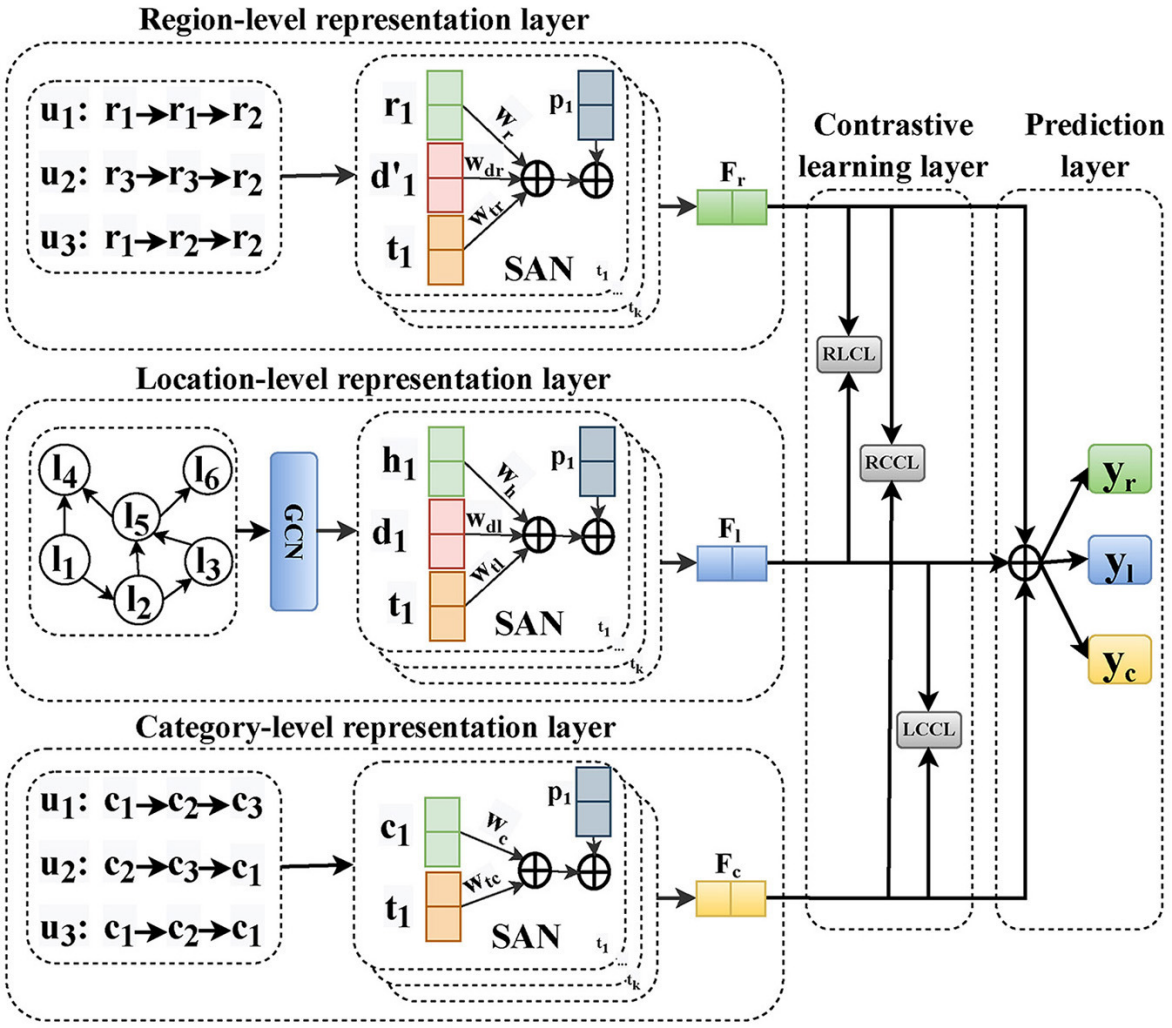
Illustration of the MGCL model that includes location-level, region-level, and category-level representation layers, followed by the contrastive learning and prediction layer.

In this section, we elaborate on the proposed model, multi-granularity contrastive learning (MGCL), for next POI recommendation. The overall representation of our MGCL framework is shown in Figure 2. Specifically, MGCL has the following parts: (1) Location-level representation layer aims to capture global across-user and local individual sequential transition patterns through location granularity sequences. (2) Region-level representation layer aims to learn about the sequential transition patterns based on region granularity sequences of local individual users. (3) Category-level representation layer aims to learn about the sequential transition patterns based on category granularity sequences of local individual users. (4) Contrastive learning layer aims to capture the collaborative signals between different granularities and enable POI representation to achieve high-quality representation by multi-granularity modeling. (5) The prediction layer aims to predict the next POI. We will introduce each layer in detail.

### 4.1 Location-level representation layer

This layer aims to capture global across-user and local individual sequential transition patterns through location granularity sequences.

#### 4.1.1 POI representation via GCN

At first, we generate a directed graph of POI according to the check-in trajectories of all users, which can model the sequential pattern of all users globally, and then capture the collaborative signal across users. After getting the constructed directed POI graph, we use GCN to obtain the POI representation,


(1) $$\begin{array}{l} {\text{H}}^{\left(z+1\right)}=ReLU\left({\tilde{D}}^{-1}\tilde{A}{\text{H}}^{\left(z\right)}{\text{W}}^{\left(z\right)}\right), \end{array}$$

Here, *ReLU* denotes the activation function, $\tilde{\text{A}}=\text{A}+\text{I}$; **A**∈ℝ^|ℒ| × |ℒ|^ is the in-degree adjacency matrix; **I** is the identity matrix representing the self-connection of each node; $\tilde{\text{D}}\in {\mathbb{R}}^{\left|ℒ|\times |ℒ\right|}$ is the diagonal in-degree matrix with $\tilde{\text{D}}ii=\underset{j}{\sum }{\tilde{\text{A}}}_{ij}$; **H**^(*z*)^∈ℝ^|ℒ| × *d*^ is the POI embedding matrix in the *z*-th layer; *d* is the embedding size; **H**^(0)^ is the initialized POI embedding matrix; **W**^(*z*)^∈ℝ^*d*×*d*^ is a layer-wise trainable weight matrix.

#### 4.1.2 Location-level POI representation

The goal of the next POI recommendation is to predict for a single user where to go in the next time. Therefore, the location-level, that is, the POI check-in sequence within a user, also plays a crucial role in user preference modeling. To this end, we model local user sequential transition patterns with the self-attention network (SAN). On the one hand, SAN can model the context information among non-continuous check-in data and adaptively aggregate it according to the corresponding weight. On the other hand, SAN can model the context information of the current POI.

After GCN, the POI in the check-in track of user *u* is expressed as ${\text{H}}^{u}=\left[{\text{h}}_{t_{1}}^{u},{\text{h}}_{t_{2}}^{u},...,{\text{h}}_{t_{k}}^{u}\right]$, where **h**∈ℝ^*d*^ is the output of the last layer of GCN. To distinguish different positions of POI in the check-in trajectory, we sum the embedding of position **p** with the above POI representation. In addition, in the next POI recommendation task, the temporal and spatial context information is very important. Therefore, we use these two factors to enhance the representation of POI embedding. The enhanced POI is represented as follows:


(2) $$\begin{array}{l} {\tilde{\text{H}}}^{u}=\left[\begin{array}{c} {\text{h}}_{t_{1}}^{u}{\text{W}}_{h}+{\text{d}}_{1}^{u}{\text{W}}_{d,l}+{\text{t}}_{1}^{u}{\text{W}}_{t,l}+{\text{p}}_{1}\\ {\text{h}}_{t_{2}}^{u}{\text{W}}_{h}+{\text{d}}_{2}^{u}{\text{W}}_{d,l}+{\text{t}}_{2}^{u}{\text{W}}_{t,l}+{\text{p}}_{2}\\ ...\\ {\text{h}}_{t_{k}}^{u}{\text{W}}_{h}+{\text{d}}_{k}^{u}{\text{W}}_{d,l}+{\text{t}}_{k}^{u}{\text{W}}_{t,l}+{\text{p}}_{k} \end{array}\right], \end{array}$$

where **W** is the learnable weight matrix; ${\text{d}}_{k}^{u}\in {\mathbb{R}}^{d}$ is the representation of distance $d_{k}^{u}$ from $l_{t_{k-1}}^{u}$ to $l_{t_{k}}^{u}$; $d_{1}^{u}=0$; ${\text{t}}_{k}^{u}\in {\mathbb{R}}^{d}$ is the representation of temporal context; and ${\text{p}}_{k}\in {\mathbb{R}}^{d}$ is the position representation.

To capture the sequential dependencies at the user's local level, we input the augmented POI representation ${\tilde{H}}^{u}$ into the SAN. It is calculated as follows:


(3) $$S_{l}^{u}=softmax(\frac{({\tilde{H}}^{u}W_{l}^{Q}){\left({\tilde{H}}^{u}W_{l}^{K}\right)}^{T}}{\sqrt{d}})({\tilde{H}}^{u}W_{l}^{V}),$$

where ${\text{S}}_{l}^{u}\in {\mathbb{R}}^{k\times d}$ is the augmented representation of POI in ℒ^*u*^ through the SAN; ${\text{W}}_{l}^{Q},{\text{W}}_{l}^{K},{\text{W}}_{l}^{V}\in {\mathbb{R}}^{d\times d}$ are the query, key, and value projection matrices; and $\sqrt{d}$ to prevent the value of the input *softmax* from being too large, the partial derivative tends to approach 0.

Applying feed-forward networks (FFNs) to ${\text{S}}_{l}^{u}$ can make the model non-linear, as follows:


(4) $$F_{l}^{u}=ReLU(S_{l}^{u}W_{1}+b_{1})W_{2}+b_{2},$$

where ${\text{F}}_{l}^{u}$ is the augmented POI representation in ℒ^*u*^ through the FFN; **W** is the learnable weight matrix, and **b** is the bias vector.

### 4.2 Region-level representation layer

The purpose of this layer is to learn about the sequential transition patterns based on region granularity sequence of local individual users.

#### 4.2.1 Region-level POI representation

The sequential transformation patterns at the region-level are similar to the location-level, which are also affected by two factors, temporal and spatial. Hence it is crucial to take these two factors into account, so the enhanced regional-level preference representation **R**^*u*^ is as follows:


(5) $$R^{u}=\left[\begin{array}{c} r_{t_{1}}^{u}W_{r}+{d^{\prime }}_{1}^{u}W_{d,r}+t_{1}^{u}W_{t,r}+p_{1}\\ r_{t_{2}}^{u}W_{r}+{d^{\prime }}_{2}^{u}W_{d,r}+t_{2}^{u}W_{t,r}+p_{2}\\ \ldots \\ r_{t_{k}}^{u}W_{r}+{d^{\prime }}_{k}^{u}W_{d,r}+t_{k}^{u}W_{t,r}+p_{k} \end{array}\right],$$

where ${\text{r}}_{t_{k}}^{u}\in \text{R}$ is the representation of $R_{t_{k}}^{u}$ in ℛ^*u*^ and **R**∈ℝ^|ℛ| × *d*^ is the region representation matrix. ${d^{\prime }}_{k}^{u}\in {\mathbb{R}}^{d}$ is the representation of distance ${d^{\prime }}_{k}^{u}$ between $r_{t_{k-1}}^{u}$ and $r_{t_{k}}^{u}$; ${d^{\prime }}_{1}^{u}=0$.

We then feed **R**^*u*^ into the SAN and FFN:


(6) $$S_{r}^{u}=softmax(\frac{(R^{u}W_{\text{r}}{\;}^{Q}){\left(R^{u}W_{\text{r}}{\;}^{K}\right)}^{T}}{\sqrt{d}})(R^{u}W_{\text{r}}{\;}^{V}),$$

where ${\text{S}}_{r}^{u,i}\in {\mathbb{R}}^{d\times d}$ is the refined representation of regions in ℛ^*u*^ through the SAN. Applying FFN to ${\text{S}}_{r}^{u}$ can make the model non-linear. We can obtain ${\text{F}}_{r}^{u}$ as the refined representation of regions in ℛ^*u*^.


(7) $$F_{r}^{u}=ReLU(S_{r}^{u}W_{3}+b_{3})W_{4}+b_{4},$$

where ${\text{F}}_{r}^{u}$ is the enhanced representation in ℛ^*u*^ through the FFN.

### 4.3 Category-level representation layer

The purpose of this layer aims to learn about the sequential transition patterns based on category granularity sequence of local individual users.

#### 4.3.1 Category-level POI representation

Category information can reflect the user's intention to a certain extent, and the change in the POI category represents the dynamic shift in the user's intention. Similarly, it also has an obvious sequential transition pattern, and the sequential changes at the category-level are affected by the time factor. Hence it is crucial to consider this factor. Therefore, the augmented representation of the categories sequences **C**^*u*^ is as follows:


(8) $$\begin{array}{l} {\text{C}}^{u}=\left[\begin{array}{c} {\text{c}}_{t_{1}}^{u}{\text{W}}_{c}+{\text{t}}_{1}^{u}{\text{W}}_{t,c}+{\text{p}}_{1}\\ {\text{c}}_{t_{2}}^{u}{\text{W}}_{c}+{\text{t}}_{2}^{u}{\text{W}}_{t,c}+{\text{p}}_{2}\\ ...\\ {\text{c}}_{t_{k}}^{u}{\text{W}}_{c}+{\text{t}}_{k}^{u}{\text{W}}_{t,c}+{\text{p}}_{k} \end{array}\right], \end{array}$$

where ${\text{c}}_{t_{k}}^{u}\in \text{C}$ is the representation of $C_{t_{k}}^{u}$ in 𝒞^*u*^ and **C**∈ℝ^|𝒞| × *d*^ is the category representation matrix.

We then feed **C**^*u*^ into the SAN and FFN:


(9) $$S_{c}^{u}=softmax(\frac{(C^{u}W_{\text{c}}{\;}^{Q}){\left(C^{u}W_{\text{c}}{\;}^{K}\right)}^{T}}{\sqrt{d}})(C^{u}W_{\text{c}}{\;}^{V}),$$

where ${\text{S}}_{c}^{u}\in {\mathbb{R}}^{d\times d}$ is the refined representation of category in 𝒞^*u*^ through the SAN. Applying FFN to ${\text{S}}_{c}^{u}$ can make the model non-linear.


(10) $$F_{c}^{u}=ReLU(S_{c}^{u}W_{5}+b_{5})W_{6}+b_{6},$$

where ${\text{F}}_{c}^{u}$ is the refined representations of categories in 𝒞^*u*^ through the FFN.

### 4.4 Contrastive learning layer

To facilitate the transfer of patterns across multiple granularities, we introduce a contrastive learning approach that conducts contrastive learning for Point-of-Interest (POI) representation across any two granularities.

#### 4.4.1 Contrastive learning

Following Sections 4.1, 4.2, and 4.3, we can create three granularity POI representation based on location-level, region-level, and category-level sequences. The key step in contrastive learning is to select high-quality positive sample pairs and negative sample pairs. In most cases, positive sample pairs emphasize the consistency of the same item in different views, while negative sample pairs focus more the inconsistency between different items. In this study, we select the same POI representation from the different granularities as positive samples. We select the different POI representations from the mini-batch as negative samples. Once positive and negative sample pairs are identified, we employ the InfoNCE (Noise-Contrastive Estimation) contrast loss function to maximize the consistency of positive sample pairs and minimize the consistency between negative sample pairs. The specific formulation of the contrastive loss function is as follows:


(11) $$\begin{array}{l} {ℒ}_{lr}=-\sum_{u=1}^{\left|U\right|}\text{log}\\ \frac{\text{exp}(\text{sim}(F_{l}^{u},F_{r}^{u}))}{\text{exp}(\text{sim}(F_{l}^{u},F_{r}^{u}))+\sum_{u^{-}\in N^{-}}\text{exp}(\text{sim}(F_{l}^{u},F_{l}^{u^{-}})))}, \end{array}$$

(12) $$\begin{array}{l} {ℒ}_{lc}=-\sum_{u=1}^{\left|U\right|}\text{log}\\ \frac{\text{exp}(\text{sim}(F_{l}^{u},F_{c}^{u}))}{\text{exp}(\text{sim}(F_{l}^{u},F_{c}^{u}))+\sum_{u^{-}\in N^{-}}\text{exp}(\text{sim}(F_{l}^{u},F_{l}^{u^{-}})))}, \end{array}$$

(13) $$\begin{array}{l} {ℒ}_{rc}=-\sum_{u=1}^{\left|U\right|}\text{log}\\ \frac{\text{exp}(\text{sim}(F_{r}^{u},F_{c}^{u}))}{\text{exp}(\text{sim}(F_{r}^{u},F_{c}^{u}))+\sum_{u^{-}\in N^{-}}\text{exp}(\text{sim}(F_{r}^{u},F_{r}^{u^{-}})))}, \end{array}$$

where sim(·) is the cosine similarity function, |*U*| is the number of users. ℒ_*lr*_, ℒ_*lc*_, and ℒ_*rc*_ are location-level and region-level contrastive learning loss functions, location-level and category-level contrastive learning loss functions, and region-level and category-level contrastive learning loss functions, respectively. 𝒩^−^ is the set of negative sample pairs within the mini-batch.

Ultimately, contrastive learning by minimizing the loss function as follows:


(14) $$\begin{array}{l} {ℒ}_{cl}={ℒ}_{lr}+{ℒ}_{lc}+{ℒ}_{rc}. \end{array}$$

### 4.5 Prediction layer

In this layer, we integrate the representations of multi-granularity as the final POI representation, and the user preferences are summarized as follows:


(15) $$\begin{array}{l} {\text{f}}_{t_{k}}={\text{f}}_{t_{k}}^{l}+{\text{f}}_{t_{k}}^{r}+{\text{f}}_{t_{k}}^{c}, \end{array}$$

where ${\text{f}}_{t_{k}}^{l},{\text{f}}_{t_{k}}^{r},{\text{f}}_{t_{k}}^{c}\in {\text{F}}_{l}^{u},{\text{F}}_{r}^{u},{\text{F}}_{c}^{u}$, which are the representations of multi-granularity.

The POI representation at time *t*_*k*_ is given, we can predict the next POI by:


(16) $$\begin{array}{l} {ŷ}_{t_{k+1}}^{l}=softmax\left({\text{H}}^{\left(\text{Z}\right)}{\left({\text{f}}_{t_{k}}\right)}^{⊤}\right), \end{array}$$

where ${ŷ}_{t_{k+1}}^{l}$ represents the scores over all candidate POI, and *Z* is the number of GCN layers.

Therefore, the loss function for the next POI prediction is calculated as follows:


(17) $${ℒ}_{l}=-\sum log({\hat{y}}_{t_{k+1}}^{l})+(1-{\hat{y}}_{t_{k+1}}^{l})log({\hat{y}}_{t_{k+1}}^{l}),$$

where $y_{t_{k+1}}^{l}$ is the one-hot vector of the ground-truth POI *l*_*t*_*k*+1__ at time *t*_*k*+1_.

Meanwhile, the two auxiliary tasks to predict the next region and category:


(18) $$\begin{array}{l} {ŷ}_{t_{k+1}}^{r}=softmax\left(\text{R}{\left({\text{f}}_{t_{k}}^{r}\right)}^{⊤}\right),{ŷ}_{t_{k+1}}^{c}=softmax\left(\text{C}{\left({\text{f}}_{t_{k}}^{c}\right)}^{⊤}\right), \end{array}$$

where ${ŷ}_{t_{k+1}}^{r}$ are the prediction scores of all candidate regions; where ${ŷ}_{t_{k+1}}^{c}$ are the prediction scores of all candidate categories.

Therefore, the loss functions of the two auxiliary tasks are as follows:


(19) $${ℒ}_{r}=-\sum log({\hat{y}}_{t_{k+1}}^{r})+(1-{\hat{y}}_{t_{k+1}}^{r})log({\hat{y}}_{t_{k+1}}^{r}),$$

(20) $${ℒ}_{c}=-\sum log({\hat{y}}_{t_{k+1}}^{c})+(1-{\hat{y}}_{t_{k+1}}^{c})log({\hat{y}}_{t_{k+1}}^{c}),$$

where ${\text{y}}_{t_{k+1}}^{r}$ are one-hot vectors of the ground truth region *r*_*t*_*k*+1__ at time *t*_*k*+1_; ${\text{y}}_{t_{k+1}}^{c}$ are one-hot vectors of the ground truth region *c*_*t*_*k*+1__ at time *t*_*k*+1_.

Ultimately, the recommendation task loss function is defined as:


(21) $$\begin{array}{l} {ℒ}_{npr}={ℒ}_{l}+{ℒ}_{r}+{ℒ}_{c}. \end{array}$$

Finally, we jointly optimize the above tasks as below:


(22) $$\begin{array}{l} ℒ={ℒ}_{npr}+\lambda {ℒ}_{cl}. \end{array}$$

where λ is the weight to balance the two tasks loss.

### 4.6 Time complexity

Time complexity refers to the time it takes to execute an algorithm. The item graph is the largest graph, and the time complexity is the upper limit. The time consumption of our model is primarily attributed to two main components. The first component constructs the multi-dimensional graph embedding layer. Constructing the global graph requires traversing each edge, which has a time complexity of *O*(*E*). Calculating the edge weights in the graph takes *O*(*I*^2^), and the GCN computation also takes *O*(*I*^2^). The second component involves the temporal self-attention layer. For each channel, updating the item embedding has a time complexity of *O*(*n*^2^*d*). Consequently, the overall time complexity of our model is *O*(*E*+*I*^2^+*I*^2^+*n*^2^*d*).

## 5 Experiments

In this section, we initially provide a comprehensive overview of the dataset, the baseline models employed for comparison, the evaluation metrics applied in the experiments, and the specifics of our experimental setup. Subsequently, we showcase and analyze the experimental results of our model in comparison to the baseline model, delving into the primary reasons behind these outcomes. Following this, we undertake an ablation study on the key components of our model. Lastly, a detailed analysis is performed on the main parameters within the MGCL model. To address specific inquiries, we conduct experiments aimed at providing insights into the following questions:

**RQ1:** Can MGCL demonstrate superior performance compared to the baseline models in the next Point of Interest (POI) recommendation task?
**RQ2:** What impact do the various components of MGCL have on its overall performance?
**RQ3:** How does the performance of MGCL vary with changes in the key hyperparameters?

### 5.1 Datasets

To assess the recommendation efficacy of MGCL, we opt for experimentation on three real-world public datasets. The selected datasets include Singapore (SIN), Foursquare check-in records from Calgary (CAL), and Phoenix (PHO). For each user, the check-in records are temporally partitioned by day and subsequently organized in the chronological order. The dataset is then divided into training, validation, and test sets with a ratio of 8:1:1. Additionally, users with fewer than three interactions in the dataset are excluded. Table 1 provides detailed information on these three public datasets.

**Table 1:** Dataset statistics.

**Dataset**	**CAL**	**PHO**	**SIN**
Users	435	2,946	8,648
POI	3,013	7,247	33,712
Check-ins	13,911	47,980	355,337
Categories	293	344	398
Regions	9	9	9
Density	1.06%	0.22%	0.12%

### 5.2 Baselines

We conducted comparisons between MGCL and the following models:

**(1) POP** relies on item popularity, suggesting items to users by arranging them based on their popularity.
**(2) BPR** (Rendle et al., 2009) is a personalized ranking algorithm grounded in Bayesian inference. It is employed in recommendation systems to acquire insights into user preferences regarding items, specifically in terms of their ranking order.
**(3) ST-RNN** (Liu et al., 2016) is an approach that employs recurrent neural networks to model and predict spatio-temporal data, capturing both spatial and temporal dependencies on the information.
**(4) ATST-LSTM** (Huang et al., 2021) is a next POI prediction model that embeds temporal and spatial information multi-modally.
**(5) SASRec** (Kang and McAuley, 2018) is a sequential recommendation model that uses self-attention networks.
**(6) LightGCN** (He et al., 2020) is a collaborative filtering recommendation model that does not use item feature information and non-linear activation.
**(7) SGRec** (Li et al., 2021a) is a next POI prediction model that uses GAT to capture sequential transition patterns of global all users and local single users.
**(8) DuoRec** (Qiu et al., 2022) engages in contrastive learning at the model level as a strategy to alleviate the degradation of representation.
**(9) MCARNN** (Liao et al., 2018) is a multi-task learning framework that leverages both next Point-of-Interest (POI) prediction and next activity prediction to enhance overall prediction performance.
**(10) iMTL** (Zhang et al., 2020a) considers spatial, temporal, POI category information and multi-task for next POI prediction.
**(11) MCMG** (Sun et al., 2022) is a next POI prediction framework with multi-granularity information and multi-task.

### 5.3 Evaluation metrics

To showcase the comprehensive effectiveness of our model, we employ two widely used evaluation metrics in next Point-of-Interest (POI) prediction, namely, Hit Ratio (**HR**) and Normalized Discounted Cumulative Gain (**NDCG**), with *K* = {5, 10}. **HR** assesses the accuracy of the recommendations, while **NDCG** is a position-aware metric that assigns greater weights to higher positions.

**HR** is used to measure whether the recommendation system successfully hits the items that the user actually likes in the candidate recommendation list given by the user. **HR** is usually defined as follows:


(23) $$\begin{array}{l} \text{HR}@K=\frac{1}{M}\overset{M}{\underset{i=1}{\sum }}\text{hits}\left(i\right), \end{array}$$

where *M* represents the number of users. hits(*i*) indicates whether the predicted item for the *i*-th user is among the top-*K* items, taking a value of 1 if it is and 0 otherwise. **HR** underscores the precision of model recommendations, with a higher value indicating better performance.

**NDCG** is an indicator used to evaluate the performance of recommendation systems. It takes into account the ranking information of items in the recommendation list and the user's preference for the items. NDCG is defined as follows:


(24) $$\begin{array}{l} \text{DCG}@k=\overset{k}{\underset{i=1}{\sum }}\frac{2^{{rel}_{i}}-1}{\text{lo}{\text{g}}_{2}\left(i+1\right)}, \end{array}$$

(25) $$\begin{array}{l} \text{IDCG}@k=\overset{k}{\underset{i=1}{\sum }}\frac{2^{{rel}_{sorted_{i}}}-1}{\text{lo}{\text{g}}_{2}\left(i+1\right)}, \end{array}$$

(26) $$\begin{array}{l} \text{NDCG}@k=\frac{\text{DCG}@k}{\text{IDCG}@k}, \end{array}$$

**DCG@k** represents the cumulative gain of items in the first *k* positions in the recommendation list. *rel*_*i*_ is the user's preference for the *i*-th item, usually using a binary flag (for example, 1 means the user likes it, 0 means he does not like it) or a real value to represent the user's preference for the item. **IDCG@k** represents the cumulative gain of items in the first *k* positions under ideal circumstances. It is the cumulative gain after the ideal ranking of the user's true preferences. **NDCG@k** is the normalized value between **DCG@k** and **IDCG@k**, which is used to compare the evaluation results of different recommendation lists. The purpose of normalization is to eliminate the impact of different recommendation list lengths on the evaluation results. **NDCG** takes into account the user's preference for items and the ranking information of items in the recommendation list. Therefore, compared with some simple evaluation indicators (such as **HR**), it reflects the performance of the recommendation system more comprehensively.

### 5.4 Parameter settings

In this study, the value of our learning rate is set at 0.0001 and the value of the training batch is 512. The embedding size for CAL dataset is 180, the embedding sizes for PHO dataset and SIN dataset are 120. The contrastive learning of weight hyper-parameter λ is searched from 0 to 1.0 with step size 0.02; The number of heads *n*_*h*_, blocks *n*_*b*_ for SAN, and the number of layers *n*_*l*_ for GCN are searched in {1, 2, 3, 4}.

### 5.5 Performance comparison (**RQ1**)

To validate the overall performance of the MGCL model, we conducted a comparison with state-of-the-art recommendation methods. The results are presented in Table 2. Based on the table, the following conclusion can be drawn:

**Table 2:** Comparisons between three datasets.

**Methods**	**CAL**				**PHO**				**SIN**			
	**HR@5**	**HR@10**	**NDCG@5**	**NDCG@10**	**HR@5**	**HR@10**	**NDCG@5**	**NDCG@10**	**HR@5**	**HR@10**	**NDCG@5**	**NDCG@10**
POP	0.0622	0.0913	0.0375	0.0463	0.016	0.0223	0.0114	0.0131	0.0125	0.0293	0.0106	0.0151
BPR	0.0862	0.1046	0.0467	0.0793	0.0487	0.0585	0.0256	0.0304	0.0352	0.0525	0.0222	0.0380
ST-RNN	0.1469	0.1731	0.1225	0.1508	0.1240	0.2028	0.0802	0.1229	0.0959	0.1370	0.0655	0.0794
ATST-LSTM	0.2027	0.2898	0.1684	0.2236	0.1579	0.2377	0.1033	0.1385	0.1296	0.1933	0.1027	0.1476
SASRec	0.3077	0.4108	0.2646	0.2723	0.2807	0.3325	0.2021	0.2101	0.2301	0.2885	0.1301	0.1524
LightGCN	0.2954	0.3731	0.1868	0.2076	0.2563	0.3151	0.1881	0.2194	0.2165	0.2691	0.1263	0.1335
SGRec	0.3879	0.4854	0.2894	0.3112	0.2897	0.3401	0.2048	0.2249	0.2310	0.2953	0.1530	0.1739
DuoRec	0.3311	0.4503	0.2386	0.2777	0.2464	0.3315	0.1566	0.1789	0.2329	0.3254	0.1617	0.1914
MCARNN	0.2451	0.3286	0.2015	0.2693	0.1905	0.2726	0.1264	0.1617	0.2018	0.2692	0.1169	0.1591
iMTL	0.2216	0.3104	0.2031	0.2545	0.1830	0.2747	0.1301	0.1632	0.1505	0.1801	0.1051	0.1423
MCMG	0.4426	0.5333	0.3431	0.3743	0.3027	0.3843	0.2211	0.2489	0.2498	0.3338	0.1729	0.1987
CLMG	**0.5166**	**0.6093**	**0.3946**	**0.4114**	**0.3496**	**0.4275**	**0.2520**	**0.2742**	**0.2668**	**0.3564**	**0.1881**	**0.2169**
Improve	16.72%	14.25%	15.01%	9.91%	15.49%	11.24%	13.98%	10.16%	6.81%	6.77%	8.79%	9.16%

The bold values represent the results obtained in this study, while the italicized values represent the best baseline results.

The POP and BPR models are classic non-sequential models, which have achieved the worst recommendation effect on all datasets, and the main reason is that they do not have the order information of the modeled sequence.

ATST-LSTM and ST-RNN models are RNN-based, which achieved better experimental performance than classic non-sequential models because they can effectively model the sequential transition patterns of users. SASRec achieves better experimental results than the above models in most cases. This is because the SAN can effectively capture contextual information while capturing sequential transition patterns. LightGCN and SGRec are GNN-based models that consider the global POI check-in trajectories. It demonstrates the effectiveness of GNNs in capturing global cross-user high-order information. SGRec achieves strong experimental results by fusing POI category information.

DuoRec is a CL-based model that shows better performance compared to SASRec. This may be because contrastive learning, as a regularization objective, can deal with the data sparsity issue and improve the performance of the model. MCARNN, iMTL, and MCMG are MTL-based models that achieve strong experimental results, which demonstrate the positive effect of multi-task prediction on the next POI prediction task.

MGCL demonstrates superior performance across all three datasets when compared to all baseline models, showcasing a relative improvement ranging from 6 to 15%. This notable enhancement in performance can be attributed to several key factors: First, the adoption of multi-granularity modeling proves advantageous as it enables the model to effectively capture sequential patterns at various levels of granularity. This approach allows for a more nuanced understanding of the underlying data structures, leading to improved predictive capabilities. Second, the integration of contrastive learning within the model addresses the challenge of data sparsity, contributing to enhanced robustness. Contrastive learning mechanisms facilitate effective learning even in scenarios with limited data, thereby improving the model's ability to generalize and make accurate predictions. Finally, the implementation of multi-task learning proves beneficial for the primary task. By jointly training the model on multiple related tasks, the shared knowledge and representations contribute to improved performance on the main task of next POI recommendation. This collaborative learning approach enhances the overall effectiveness of the model by leveraging complementary information from different tasks. In summary, the success of MGCL can be attributed to its multi-faceted approach, combining multi-granularity modeling, contrastive learning, and multi-task learning to address specific challenges in the recommendation task, resulting in substantial performance gains across diverse datasets.

### 5.6 Ablation study (**RQ2**)

In this section, we conduct an ablation study on the key components of our framework. Tables 3–5 present the performance of the MGCL model and its variants, which fall into three main categories:

**Table 3:** Performance of the CL-based variants.

**Methods**	**CAL**				**PHO**				**SIN**			
	**HR@5**	**HR@10**	**NDCG@5**	**NDCG@10**	**H@5**	**H@10**	**NDCG@5**	**NDCG@10**	**HR@5**	**HR@10**	**NDCG@5**	**NDCG@10**
MGCL	0.5166	0.6093	0.3946	0.4114	0.3496	0.4275	0.2520	0.2742	0.2668	0.3564	0.1881	0.2169
MGCL-*gccl*	0.4901	0.6093	0.3921	0.4173	0.3351	0.4203	0.2215	0.2486	0.2613	0.3508	0.1812	0.2100
MGCL-*grcl*	0.4901	0.5695	0.3679	0.3928	0.3315	0.4239	0.2272	0.2511	0.2649	0.3518	0.1848	0.2121
MGCL-*crcl*	0.5166	0.5828	0.3864	0.4085	0.3279	0.4221	0.2079	0.2399	0.2598	0.3497	0.1809	0.2106
MGCL-*cl*	0.4426	0.5333	0.3431	0.3743	0.3027	0.3843	0.2211	0.2489	0.2498	0.3338	0.1729	0.1987

**(1) CL-based variants:** This section aims to verify the contribution of contrastive learning to the MGCL method.

- *MGCL-lccl:* Removing the contrastive learning component between location-level and category-level POI representation.
- *MGCL-lrcl:* Removing the contrastive learning component between location-level POI representation and region-level POI representation.
- *MGCL-crcl:* Removing the contrastive learning component between category-level POI representation and region-level POI representation.
- *MGCL-cl:* Removing the contrastive learning component.

The results are shown in Table 3. From the table, we can draw the following conclusions: First, MGCL-*cl* has the worst performance compared to other variants, which proves that contrastive learning plays an important role in the next POI recommendation. Since contrastive learning can capture collaborative signals between multi-granularity and facilitate mutual enhancement, the model can obtain a higher-quality POI representation. Second, removing different contrastive learning components, all achieve varying degrees of decline relative to our MGCL model. MGCL-*lccl* can dig out cooperative signals between location-level and category-level sequences, MGCL-*lrcl* can dig out cooperative signals between location-level and region-level sequences, MGCL-*crcl* can mine category-level sequences and region-level co-signaling between sequences. When the above components are removed separately, the effect of the model is reduced to varying degrees. From the experimental results, we can see that contrastive learning between any two granularities can enhance the POI representation.

**(2) MG-based variants:** This subsection aims to verify the contribution of different granularities of information to the MGCL model. (When verifying the importance of multi-granularity information, the contrastive learning module is also removed.)

- *MCMG-cl, c:* Removing the category-level component and using region-level and location-level components for the next POI recommendation;
- *MCMG-cl, r:* Removing the region-level component and using location-level and category-level components for the next POI recommendation;
- *MCMG-cl, cr:* Removing the category-level and region-level components and using the location-level component for the next POI recommendation.

From Table 4: the variant approach shows the weakest recommendation performance when both category-level and region-level components are excluded. Within the various configurations, MGCL-*cl, cr* stands out with a more significant decline in performance compared to MGCL-*cl, c* and MGCL-*cl, r*, highlighting the vital roles played by both region and category modeling. It is important to note the superior performance of MGCL-*cl, c* over MGCL-*cl, r*, indicating that the accuracy of next POI recommendations relies more heavily on region information than on category information. This underscores the importance of considering geographical context in refining recommendation systems for POI.

**Table 4:** Performance of the MG-based variants.

**Methods**	**CAL**				**PHO**				**SIN**			
	**HR@5**	**HR@10**	**NDCG@5**	**NDCG@10**	**H@5**	**H@10**	**NDCG@5**	**NDCG@10**	**HR@5**	**HR@10**	**NDCG@5**	**NDCG@10**
MGCL	0.5166	0.6093	0.3946	0.4114	0.3496	0.4275	0.2520	0.2742	0.2668	0.3564	0.1881	0.2169
MGCL-*c, cl*	0.4095	0.4935	0.3216	0.3509	0.1681	0.2135	0.1781	0.2006	0.2230	0.2980	0.1454	0.1672
MGCL-*r, cl*	0.3878	0.4673	0.3168	0.3457	0.1030	0.1308	0.0551	0.0621	0.2072	0.2769	0.1342	0.1543
MGCL-*cr, cl*	0.1872	0.2256	0.1132	0.1235	0.0651	0.0827	0.0385	0.0434	0.0900	0.1203	0.0585	0.0673

**(3) MT-based variants:** This section aims to verify the contribution of multi-tasks to the MGCL method.

- *MGCL-r*_*task*_*:* Removing the region recommendation task;
- *MGCL-c*_*task*_*:* Removing the category recommendation task;
- *MGCL-rc*_*tasks*_*:* Removing the region and category recommendation tasks.

Table 5 illustrates the performance of next POI recommendations, and the experimental findings demonstrate a consistent decline in recommendation performance when various components are removed. The model encompasses three distinct recommendation tasks, and the least favorable results in predicting the next POI emerge when both the prediction area and category tasks are excluded. Intriguingly, it is observed that omitting the prediction area task yields better recommendation performance than excluding the prediction category task. This highlights the pivotal role of the prediction area task component within our model, emphasizing its significance in achieving optimal recommendation outcomes.

**Table 5:** Performance of the MT-based variants.

	**CAL**				**PHO**				**SIN**			
	**HR@5**	**HR@10**	**NDCG@5**	**NDCG@10**	**H@5**	**H@10**	**NDCG@5**	**NDCG@10**	**HR@5**	**HR@10**	**NDCG@5**	**NDCG@10**
MGCL	0.5166	0.6093	0.3946	0.4114	0.3496	0.4275	0.2520	0.2742	0.2668	0.3564	0.1881	0.2169
MGCL-*r*_*task*_	0.4221	0.5000	0.3537	0.4390	0.2971	0.3714	0.2131	0.2342	0.2547	0.3477	0.1789	0.2089
MGCL-*c*_*task*_	0.4503	0.5298	0.3337	0.3572	0.3514	0.4366	0.2485	0.2769	0.2542	0.3413	0.1769	0.2055
MGCL-*rc*_*tasks*_	0.2649	0.3841	0.1855	0.2215	0.3062	0.3786	0.2101	0.2336	0.2340	0.3195	0.1577	0.1855

### 5.7 Parameter sensitivity analysis (**RQ3**)

**Figure 3:**
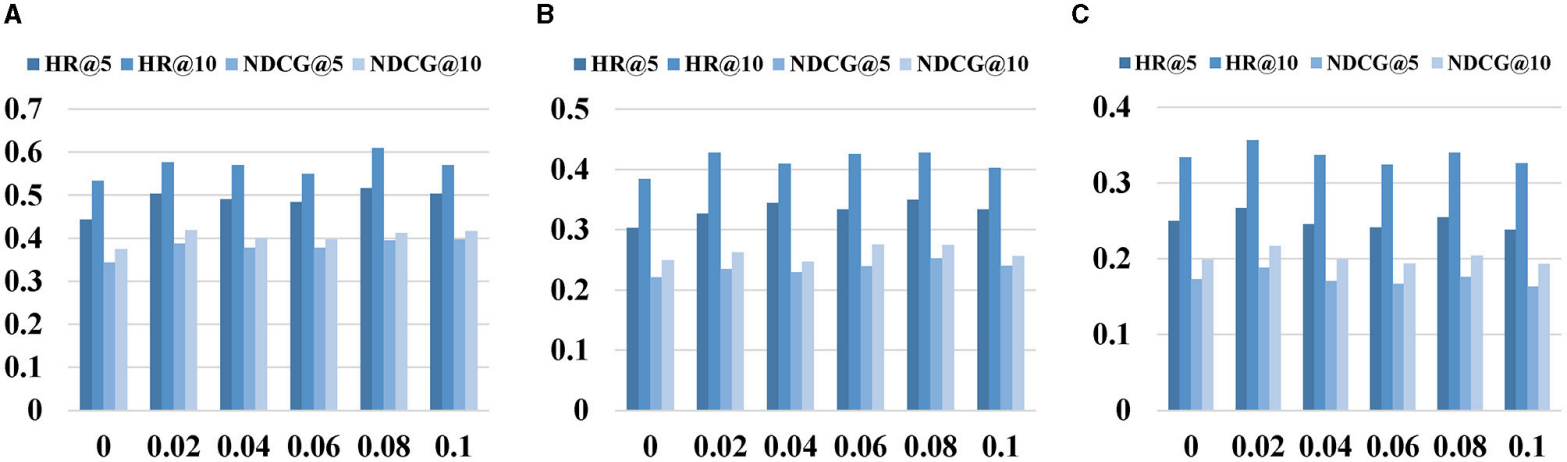
Impact of contrastive learning weight λ. **(A)** CAL. **(B)** PHO. **(C)** SIN.

**Figure 4:**
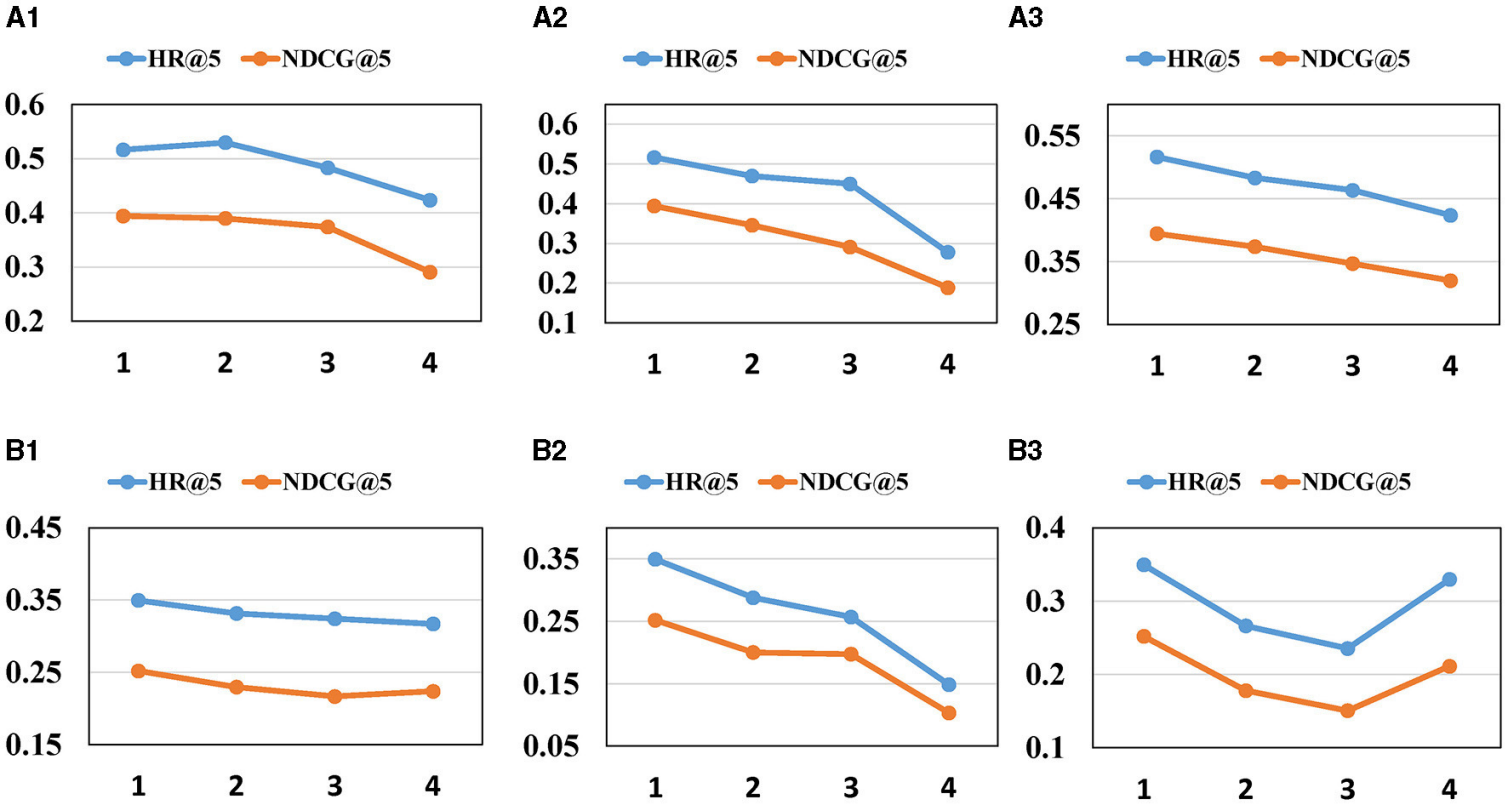
Impact of hyper-parameters on (HR, NDCG)@5. **(A1, B1)** The impact of heads of SA. **(A2, B2)** The impact of blocks of SA. **(A3, B3)** The layers of GCN.

**Figure 5:**
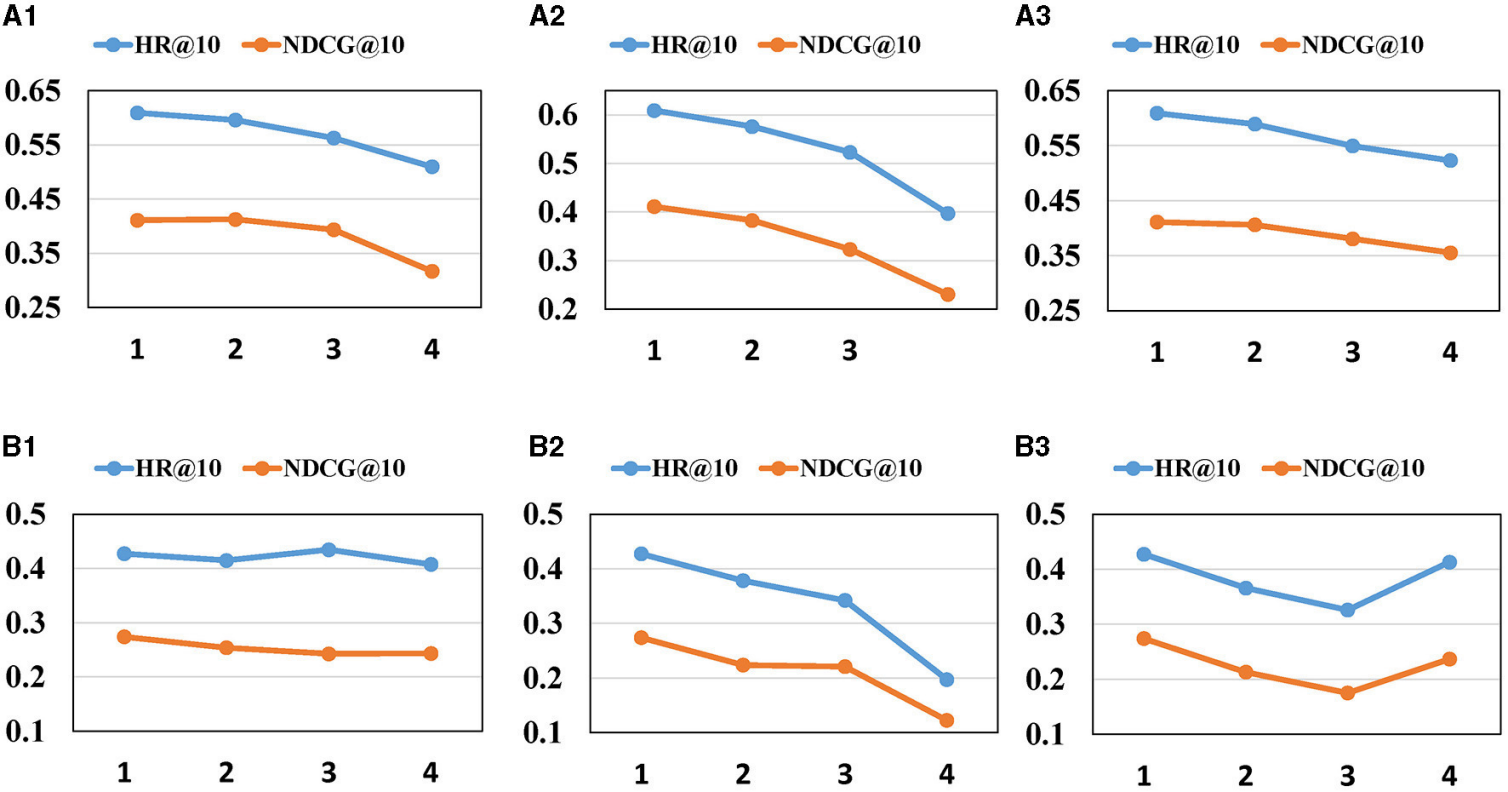
Impact of hyper-parameters on (HR, NDCG)@10. **(A1, B1)** The impact of heads of SA. **(A2, B2)** The impact of blocks of SA. **(A3, B3)** The layers of GCN.

In this section, we investigate our model's sensitivity in relation to several key hyper-parameters, Figures 3–5 depict the results of the parameter sensitivity analysis on next POI recommendation.

Figure 3 depicts the experimental results for different λ values, where λ serves as the weight controlling the intensity of contrastive learning. Its range spans from 0 to 0.1, with a step size of 0.02. The figure reveals that as the value of λ increases, the recommendation performance shows continuous improvement. However, beyond a certain threshold, the recommendation performance begins to decline. Specifically, for the CAL and PHO datasets, the optimal performance is achieved at λ = 0.08, while the SIN dataset attains the best recommendation performance at λ = 0.02. This highlights the significance of correctly choosing weight hyperparameters, as opting for values that are either too large or too small can lead to performance degradation.

Figures 4, 5 offer the following observations: (1) With the increasing number of heads and blocks in the self-attention network, the performance of MGCL gradually decreases. This phenomenon may be attributed to the cumulative error becoming larger as the attention heads and blocks increase, resulting in a decline in model performance. (2) A similar trend is observed when the number of Graph Convolutional Network (GCN) layers increases. Excessive blocks and layers can lead to the overfitting problem, contributing to the degradation of MGCL's performance.

## 6 Conclusion

In this study, we present a framework called Multi-Granularity Contrastive Learning (MGCL) for Next POI Recommendation. Our framework utilized multi-granularity and contrastive learning to improve the overall prediction accuracy. Experiment results show that MGCL significantly outperforms baseline methods. In future studies, we plan to delve deeper into enhancing the privacy aspects of our framework. Additionally, we are keen on incorporating textual information, such as users' reviews and POI attributes. Analyzing users' reviews can provide valuable insights into their preferences and sentiments, contributing to a more nuanced understanding of user behavior. Furthermore, exploring different modalities of data, such as images associated with POI or temporal patterns in user behavior, could offer new dimensions for model enhancement. Integrating these diverse data sources may lead to a more comprehensive and effective recommendation system.

## Data availability statement

The original contributions presented in the study are included in the article/supplementary material, further inquiries can be directed to the corresponding author.

## Author contributions

YZ: Conceptualization, Data curation, Investigation, Methodology, Writing – original draft, Writing – review & editing. SY: Software, Validation, Visualization, Writing – review & editing. XS: Funding acquisition, Methodology, Project administration, Resources, Software, Writing – review & editing.
